# Effects of *Bacillus thuringiensis* Genetic Engineering on Induced Volatile Organic Compounds Emission in Maize and the Attractiveness to a Parasitic Wasp

**DOI:** 10.3389/fbioe.2019.00160

**Published:** 2019-07-10

**Authors:** Hao Xu, Xiaoyi Wang, Guoliang Chi, Bingchang Tan, Jianwu Wang

**Affiliations:** ^1^Key Laboratory of Agro-Environments in Tropics, Ministry of Agriculture, South China Agricultural University, Guangzhou, China; ^2^Guangdong Provincial Key Laboratory of Eco-Circular Agriculture, South China Agricultural University, Guangzhou, China; ^3^Institute of Tropical and Subtropical Ecology, South China Agricultural University, Guangzhou, China; ^4^School of Plant Protection, Nanjing Agricultural University, Nanjing, China

**Keywords:** tritrophic interactions, leaf-chewing insects, genetically modified organism, plant-insect interactions, egg parasitoids

## Abstract

In order to control lepidopteran and coleopteran insects, the genes expressing *Bacillus thuringiensis* (*Bt*) insecticidal proteins have been transferred into crops. Ecological risk assessments of the transgenic plants have included impacts on non-target entomophagous insects, such as parasitoid wasps. Herbivore-induced plant volatiles are considered to be important defensive traits of plants because these compounds play as an important role in recruitment of natural enemies. Here, we evaluated induced volatile emissions of maize seedlings of two *Bt* cultivars (5422Bt1, event Bt11 and 5422CBCL, event Mon810), and their nearly isogenic non-*Bt* line 5422. We damaged plants mechanically and then applied with the regurgitant of *Spodoptera litura* (F.) caterpillars (Lepidoptera: Noctuidae), or treated the plants with the plant hormone jasmonic acid (JA), to trigger similar defensive responses of plants. Compared to the non-*Bt* isoline 5422 and the *Bt* maize 5422CBCL, the other *Bt* maize 5422Bt1 released more (3*E*)-4,8-dimethyl-1,3,7-nonatriene (DMNT) when they were all treated by artificial wounds and caterpillar regurgitant; and released more linalool, DMNT and (*E*)-β-farnesene when applied with JA solution. As a result, the total volatile emission of the 5422Bt1 was highest. However, the difference in volatile emission did not affect the attractiveness of the *Bt* maize plants to the egg parasitoid *Trichogramma ostriniae* Pang et Chen (Hymenoptera: Trichogrammatidae) compared to the nearly isogenic non-*Bt* plants. The variability of induced volatiles of maize cultivars derived from conventional breeding programs and transgenic methods are discussed.

## Introduction

In the last two decades, farmers around the world have rapidly adopted genetically modified (GM) crops with biotech-derived beneficial traits, e.g., herbicide tolerance and pest resistance (James, 2017). Those traits have benefited humans by increasing crop productivities and reducing environmental pollutions that can be caused by applications of chemical pesticides (Cattaneo et al., 2006; Romeis et al., 2006; Wu et al., 2008; James, 2017).

*Bt* crops are continuously expressing insecticidal proteins (δ-endotoxin), which are derived from soil bacterium *Bacillus thuringiensis* (*Bt*) Berliner. Those crops are designed to defend themselves against herbivores of Coleoptera and Lepidoptera. Expressing *Bt* proteins may change some defensive characteristics of plants to some non-target organisms. For example, several *Bt* maize cultivars and a *Bt* cotton line were found to be more susceptible to aphid damages than their respective non-*Bt* isolines in the laboratory and/or in the field (Faria et al., 2007; Hagenbucher et al., 2013).

For some entomophagous arthropods (predators and parasitoids) that feed/host on non-target insects (e.g., aphids), their population has not decreased significantly in a *Bt* crop field compared to a conventional crop field (Dutton et al., 2002, 2003; Lumbierres et al., 2011; Yao et al., 2016; Romeis et al., 2019). The field studies seem to support that *Bt* proteins are not likely to be toxic to entomophagous arthropods in the natural environment. Indeed, similar conclusions are also drawn from laboratory studies: *Bt* proteins in crops have not been reported to harm entomophagous insects when their prey/hosts are not susceptible to *Bt* toxins (i.e., *Bt*-resistant herbivores) or sap-sucking insects such as aphids that feed on plant phloem sap where *Bt* toxins are with trace amounts. For example, several parasitoid species that hosted on *Bt*-resistant *Plutella xylostella* (L.) caterpillars developed with negligible negative effects (Schuler et al., 1999, 2004; Chen et al., 2008; Liu et al., 2011). The phenomenon was further confirmed by physiological data: larvae of the endoparasitoid species *Diadegma insulare* (Cresson) were found to be exposed to a biologically active form of *Bt* proteins in the *Bt*-resistant hosts, but the survival of the parasitoids did not significantly decrease (Chen et al., 2008). In addition, with the damage by the *Bt*-resistant *P. xylostella*, the attractiveness to a few parasitoid species of *Bt* rape plants was as strong as that obtained from conventional hybrids (Schuler et al., 1999, 2003; Liu et al., 2011). When feeding on *Bt* cotton plants, bodies of the sap-sucking herbivore *Ferrisia virgata* Cockerell did not contain detectable amount of *Bt* proteins, and the survival and development of the herbivore species and its predator *Cryptolaemus montrouzieri* Mulsant were negligibly affected by the *Bt* crop (Wu et al., 2014). Therefore, *Bt* proteins in crops seem not to be poisonous to parasitoids and predators, which indicates releasing natural enemies is still likely to be an effective way to control non-target or *Bt*-resistant pests in *Bt* fields (Romeis et al., 2019).

In maize, transformations of foreign genes may quantitatively change the emission of the herbivore-induced plant volatiles (HIPVs), which undertake several ecological functions including attracting natural enemies of herbivores (Heil, 2014). The *Bt* maize plant (N4640Bt, event Bt11) emitted a few volatile compounds in a smaller amount than did its isogenic non-*Bt* line when they were both treated by mechanical injury and then applied with herbivore regurgitant, possibly because the *Bt* maize allocated some resources on the biosynthesis of *Bt* proteins, and as a result, less resources to produce HIPVs (Turlings et al., 2005). The differences did not affect their attractiveness to two endoparasitoid species (Turlings et al., 2005). *Bt* transgenic events do not necessarily result in a shift of HIPVs in maize. For example, the *Bt* maize plant (DKC61-25, event Mon810) emitted similar amounts of HIPVs with its isogenic non-*Bt* line when they were damaged by the same controlled method, artificial wounds and caterpillar regurgitant (Dean and De Moraes, 2006). Since GM maize plants with *Bt* genes have been adopted on a large-scale worldwide, the effects of different transgenic events on emissions of induced volatile organic compounds (VOCs) and tritrophic interactions with different insect species need long-term evaluations.

The tobacco cutworm *S. litura* is widespread throughout tropical and subtropical Asia. Larvae of this insect feed on a wide spectrum of agricultural and horticultural crops and have caused severe damage (Wei et al., 2004). Chemical pesticides are not sufficient to control this species on some crops because larval resistance develops quickly, and as a result, biological controls with parasitoid wasps possibly act as an important alternative (Kuhar et al., 2004; Wei et al., 2004). The egg parasitoid *T. ostriniae*, endemic to China, is an important candidate to control several lepidopteran pests, such as the European corn borer (Hoffmann et al., 1995; Kuhar et al., 2004; Gardner et al., 2007). Moth eggs of many species in the Noctuidae, Pyralidae, and Plutellidae of the Lepidoptera have experienced high levels of parasitism by the parasitoids (Hoffmann et al., 1995). *S. litura* is one of the host species of the parasitic wasps (Kuhar et al., 2004). Furthermore, caterpillar-damaged plants are commonly reported to attract egg parasitoids (Reddy et al., 2002; Peñaflor et al., 2011b; War et al., 2016; Michereff et al., 2019; Ortiz-Carreon et al., 2019). However, our knowledge on how *Bt* transgenic events affect the host-finding behaviors of egg parasitoids is relatively limited. In this study, we compared the induced VOCs of two transgenic *Bt* maize plants (5422Bt1, event Bt11 and 5422CBCL, event Mon810), and their nearly isogenic non-*Bt* cultivar 5422 when they were treated by artificial wounds and caterpillar regurgitant, or the plant hormone jasmonic acid (JA). In addition, the attractiveness of intact and induced plants of the three cultivars to the generalist egg parasitoid wasps was also tested and compared.

## Materials and Methods

### Plants and Insects

Seeds of the two transgenic maize plants 5422Bt1 (event Bt11) and 5422CBCL (event Mon810) expressing Cry1Ab and the nearly isogenic non-*Bt* cultivar (5422) of the seed company Beck's Hybrids, Atlanta, Indiana, USA were provided by Dr. Cindy Nakatsu in the Agronomy Department of Purdue University, USA. All plants were cultivated in greenhouse (25°C, L:D = 16:8 h). Larvae of the generalist herbivore *S. litura* were reared on an artificial diet in the laboratory (Qi et al., 2000). The generalist egg parasitoid *T. ostriniae* (Hymenoptera: Trichogrammatidae) was originally provided by Guangdong Entomological Institute, Guangzhou, China. The parasitoids were reared on the eggs of *Pyrausta nubilalis* Hübner (Lepidoptera: Pyralidae), which were bought from an insect-rearing company. Some *S. litura* caterpillars (3rd instar) were reared in a plastic box (3 × 10 × 10 cm) and fed with 5422 maize leaves for 24 h, and then their regurgitant was collected by a pipette twice a day (20 μL, Eppendorf) (Turlings et al., 1993). Maize seedlings (14-day old) were treated with different methods to collect VOCs and for bioassays of the parasitoid species: plants damaged by scissors (1 cm length × 15 times on 2nd and 3rd leaves, i.e., two biggest leaves of maize seedlings) and then applied with 10 μL caterpillar regurgitant; or alternatively, the 2nd and 3rd leaves were painted with 20 μL plant hormone JA solution (4 mM), respectively. We tested plants immediately after the induction by caterpillar regurgitant, because they started to release plant volatiles shortly after the treatment (Erb et al., 2015). The plants treated by JA solution were left for about 14 h (overnight, L:D = 16:8 h) before used for the same tests, with consideration of that JA responses are likely to show an apparent increase in a few hours after an exogenous application of JA solution (Bruinsma et al., 2009).

### Olfactory Preference of Parasitoids

The preference of the parasitoid *T. ostriniae* were tested with a Y-tube olfactometer (ID = 2 cm, arm length = 16 cm). Each arm of the olfactometer was connected by a Teflon tube to an empty glass container (about 1 L) or a glass container of the same type where a treated or an intact plant was placed. A cleaned and humidified constant airflow (0.7 L/min) passed through the odor source and then entered in the olfactometer in which a naive parasitoid wasp was released. The parasitoid was considered to have made a choice when it chose one arm, walked to the odor source for more than 3 cm and stayed in that region for more than 30 s. When the wasp had made a choice or the testing time was up to 10 min (recorded as non-choice), the wasp was removed from the system, and then another naive wasp was released. To eliminate biased choices toward one arm position in each replicate, eight wasps were tested first (released one by one), and then the position of olfactometer was reversed. Then, another eight wasps were tested with the same olfactometer (released one by one). Each experiment was replicated four times (64 wasps in total).

### Headspace Volatile Sampling and Analyses

To identify and quantify the VOCs emitted by *Bt*/non-*Bt* maize plants under different treatments (caterpillar regurgitant and JA), each plant (14-day old) was put into a glass container (about 1 L) at the room temperature (25°C). VOCs were collected with Tenax filter (50 mg, 60–80 mesh, Supleco, Bellefonte, PA, USA) and the headspace air was pumped through the filter at a speed of 0.7 L/min for 4 h. A cleaned and humidified constant airflow entered the system with the same speed. After each collection, VOCs were eluted from the filters with 200 μL hexane (Sigma) five times. Then the elution was concentrated to about 500 μL with a gentle stream of nitrogen. The samples were then stored at −20°C until chemical analyses. Each experiment was replicated four times. In order to quantify VOCs, 10 μL of the internal standard, n-octane (200 ng in 10 μL hexane) was added to each sample. VOCs were analyzed with an Agilent 6890 gas chromatograph, connected with Agilent 5973 Network mass selective detector. A 2 μL aliquot of each sample was injected with splitless mode (280 °C) onto a non-polar column (HP-5 ms, 30 m, 0.25 mm ID, 0.25 μm film thickness, Agilent J&W Scientific, USA) at an initial column temperature of 50°C for 3 min, and then temperature was increased at a rate of 8°C per minute to 230 °C, and then the column temperature was held for 7.5 min. Helium at constant flow (0.9 ml/min) was used as carrier gas. Identifications of the compounds were initially carried out by mass spectrometry analysis: i.e., compounds were identified by comparing the mass spectra obtained from the samples with those from a reference database (NIST mass spectral library). Then those compounds were confirmed with authentic ones bought from Sigma-Aldrich (USA).

### Statistics

For the olfactometer data, statistical analyses were performed with SigmaPlot 14.0 (Systat Software Inc., San Jose, CA, USA) with a two-tailed *t*-test. For the quantity of VOCs, a one-way ANOVA was applied by the same software, and a Holm-Sidak *post hoc* analysis was used for pairwise comparisons. Statistical differences (*p* < 0.05) were indicated with different letters in the bar figures, and the detail statistical results were presented in Supplementary File 1.

## Results

For both *Bt* maize and non-*Bt* maize cultivars, their intact plants were not significantly more attractive to the parasitoid *T. ostriniae* than blank controls (Table 1). When treated by caterpillar regurgitant, both *Bt* maize and non-*Bt* maize plants were more attractive to the parasitoids than the blank arm and, by extension, their respective intact plants (Table 1). The strong attractiveness was still present when those plants were induced by plant hormone JA (Table 1).

**Table 1:** The attractiveness of the *Bt* maize and regular maize plants to the egg parasitoid.

	**Treatments**	**Wasp preference (%)**	**Wasp response (%)**	*P*-value (two-tailed *t*-test)^*^
Intact plants compared to an empty arm (blank control)	5422	55.3	59.4	0.275
	Empty arm	44.7		
	5422Bt1	56.1	64	0.172
	Empty arm	43.9		
	5422CBCL	52.8	56.3	0.348
	Empty arm	47.2		
Regurgitant treated plants compared to an empty arm (blank control)	5422	71.4	76.6	** <0.001**
	Empty arm	28.6		
	5422Bt1	68.6	79.7	** <0.001**
	Empty arm	31.4		
	5422CBCL	76.6	73.4	** <0.001**
	Empty arm	23.4		
JA treated plants compared to an empty arm (blank control)	5422	79.5	68.8	** <0.001**
	Empty arm	20.5		
	5422Bt1	72.9	75	**0.005**
	Empty arm	27.1		
	5422CBCL	71.7	71.9	** <0.001**
	Empty arm	28.3		
Comparisons between intact plants of different cultivars	5422	55.6	56.3	0.524
	5422Bt1	44.4		
	5422	55.0	62.5	0.11
	5422CBCL	45.0		
	5422Bt1	52.6	59.4	0.696
	5422CBCL	47.4		
Comparisons between regurgitant treated plants of different cultivars	5422	55.6	84.4	0.787
	5422Bt1	44.4		
	5422	53.7	84.4	0.155
	5422CBCL	46.3		
	5422Bt1	50.0	75.0	1
	5422CBCL	50.0		
Comparisons between JA treated plants of different cultivars	5422	51.9	84.4	0.773
	5422Bt1	48.1		
	5422	51.7	90.6	0.661
	5422CBCL	48.3		
	5422Bt1	48.3	90.6	0.617
	5422CBCL	51.7		

* *The bold P values indicate significant statistical differences (P < 0.05) between treatments*.

The *Bt* maize plants (5422Bt1 and 5422CBCL) were as attractive as their nearly isogenic non-*Bt* line (5422) to the parasitoid *T. ostriniae*, when they were all undamaged, applied with caterpillar regurgitant, or treated by JA (Table 1). The attractiveness to the parasitoid species of the 5422Bt1 was not significantly different from 5422CBCL once they were treated in the same way (Table 1).

**Figure 1:**
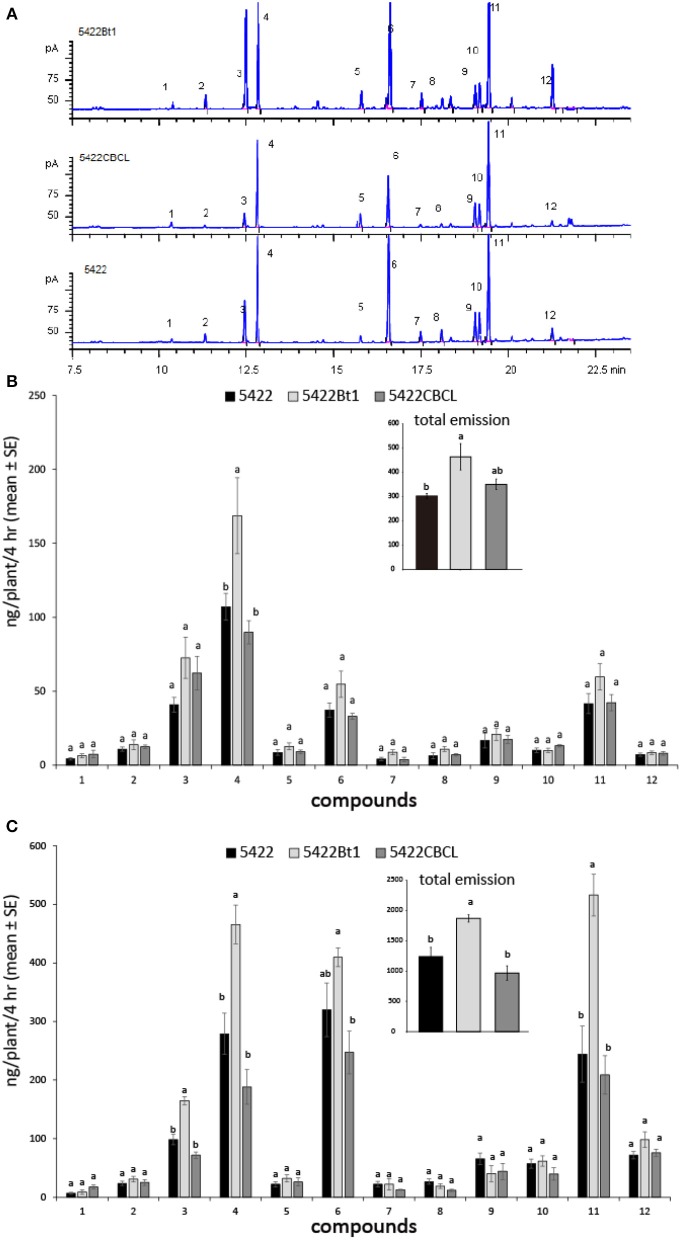
Volatile emissions of the *Bt* maize and the non-*Bt* maize lines under different treatments. **(A)** The chromatographs of maize seedlings induced by JA solution. The compounds were 1 = (*Z*)-3-hexen-1-yl acetate; 2 = (*E*)-β-ocimene; 3 = linalool; 4 = (3*E*)-4,8-dimethyl-1,3,7-nonatriene (DMNT); 5 = phenethyl acetate; 6 = indole; 7 = methyl anthranilate; 8 = geranyl acetate; 9 = (*E*)-β-caryophyllene; 10 = (*E*)-α-bergamotene; 11 = (*E*)-β-farnesene; 12 = (*E*)-nerolidol. **(B)** The volatile emissions (*N* = 4) of the three cultivars when the plants were mechanically damaged and then treated by caterpillar regurgitant. **(C)** The volatile emissions (*N* = 4) of the three cultivars when the plants were treated by JA. A one-way ANOVA with the Holm-Sidak *post hoc* analysis was used for pairwise comparisons, and letters on the bar figures indicated statistical differences (*P* < 0.05). The details of statistical results and data were presented in Supplementary Files 1, 2, respectively.

With the treatments of caterpillar regurgitant and JA, all three cultivars released 11 main volatile compounds: (*Z*)-3-hexen-1-yl acetate, (*E*)-β-ocimene, linalool, (3*E*)-4,8-dimethyl-1,3,7-nonatriene (DMNT), phenethyl acetate, indole, methyl anthranilate, geranyl acetate, (*E*)-β-caryophyllene, (*E*)-α-bergamotene, (*E*)-β-farnesene, and (*E*)-nerolidol (Figure 1A). When induced by caterpillar regurgitant, the 5422Bt1 released more DMNT than did 5422CBCL and the non-*Bt* line 5422, which probably resulted in the total volatile emission of 5422Bt1 was also highest (Figure 1B). Comparable results occurred when plants were induced by JA: 5422Bt1 released more total VOCs than did 5422 and 5422CBCL; and 5422Bt1 emitted more amounts of linalool, DMNT, and (*E*)-β-farnesene than did 5422 and 5422CBCL (Figure 1C). In addition, intact plants of the three cultivars released a few compounds, e.g., linalool, DMNT and (*E*)-β-farnesene, in trace amounts (Supplementary File 2).

## Discussion

### Genetic Transformations Sometimes Change Induced VOCs Emissions of Plants and the Possible Mechanism

In this study, we found the 5422Bt1 released a few terpenes in a higher amount than did its nearly isogenic non-*Bt* maize when the plants were treated by caterpillar regurgitant or JA. Although a few studies reported that introduction of new genes into plants did not change the VOCs emission (Dean and De Moraes, 2006; Sun et al., 2013; Liu et al., 2015), some studies confirmed that the VOCs emissions of GM plants were changed quantitatively compared to that of their regular isolines. For example, the *Bt* maize N4640Bt (event Bt11) emitted several VOCs in a smaller amount than did its isogenic non-*Bt* line when the plants were damaged by mechanical wounds and then applied with caterpillar regurgitant (Turlings et al., 2005). When infested by the leafminer species *Phyllonorycter blancardella* (Fabricius), the GM apple plants with a scab resistance gene emitted less (*E,E*)-α-farnesene than did their conventional equivalents (Vogler et al., 2010). A transgenic soybean cultivar expressing a glyphosate-resistant gene released a few volatile compounds in a higher amount than did its conventional isoline when the plants were damaged by the soybean looper *Chrysodeixis includes* (Walker) or the velvetbean caterpillar *Anticarsia gemmatalis* Hübner (Strapasson et al., 2016a,b). As a result, the herbivore-damaged GM soybean plants were more attractive to the larval parasitoid *Meteorus rubens* (Nees) than the conventional isoline (Strapasson et al., 2016a).

The mechanism of why some transformations of foreign genes in crops change some of their metabolites is unknown. However, some hypotheses have been proposed to explain different cases. For example, the quantitative modifications of inducible secondary metabolites of transgenic plants are possibly due to unintended changes on resource allocation by continuous biosynthesis of other proteins, such as *Bt*. As a result, the *Bt* maize line more likely releases several volatile compounds in a lower amount than does its non-*Bt* isoline due to deficiency of resources when the plants are induced by caterpillar regurgitant (Turlings et al., 2005). However, after genetic transformations, some inducible VOCs, such as terpenoids, have actually increased (Strapasson et al., 2016b). One possible explanation is that the newly biosynthesized protein (responsible for herbicide tolerance in this case) has affected or is involved in the plant hormone-mediated defensive pathway, which responds to produce some VOCs (Strapasson et al., 2016a). Therefore, genetic transformations possibly lead to quantitative differences in some VOCs emissions, or some other required metabolites, such as amino acids in plant phloem reported by Faria et al. (2007). The molecular mechanism needs further investigations.

### The Variation of VOCs Emission Caused by Genetic Transformations Still Fall Within the Variability Among Conventional Cultivars

The quantitative changes of induced VOCs emission that resulted from genetic transformations have been evaluated by comparing them with those from conventional cultivars. The changes caused by genetic modifications in maize and apple plants are relatively small compared to those that result from traditional breeding programs (Turlings et al., 2005; Vogler et al., 2009, 2010). For example, upon the herbivory by leafminers on apple trees, some traditional cultivars released a different amount of a key terpenoid volatile compound that attracted parasitoids, but a GM cultivar and its regular isoline emitted similar amounts of the compound, suggesting that the alterations of leaf chemistry are more apparent between conventional cultivars (Vogler et al., 2009). In maize, after analyzing 31 conventional maize lines, Degen et al. (2004) found that the variations of the total emission of induced VOCs were enormously huge, with up to a 70 times difference between two extreme lines. Some genotypes even did not produce an important terpenoid compound (*E*)-β-caryophyllene after receiving the treatment of mechanical wounds and caterpillar regurgitant (Gouinguené et al., 2001; Degen et al., 2004). The compound was reported to be a key compound involved in tritrophic interactions in the soil environment (Rasmann et al., 2005). The 5422Bt1 maize released VOCs by about 50% higher than its non-*Bt* isoline 5422. This discrepancy is probably smaller than that between many regular maize cultivars.

### Transformation of *Bt* Genes Does Not Influence the Attractiveness to Parasitoids

In our study, we found that expressing *Bt* proteins in maize plants did not affect their attractiveness to the egg parasitoid *T. ostriniae*. Egg parasitoids possibly use many kinds of volatile cues to exploit their hosts, such as host pheromones, egg odors, host frass smells or oviposition-induced plant volatiles (OIPVs) (Meiners and Hilker, 1997; Fatouros et al., 2005, 2008; Hilker and Fatouros, 2015). In addition, HIPVs are attractive to many egg parasitoids of, for example, lepidopteran and hemipteran herbivores (Reddy et al., 2002; Lou et al., 2005; Moraes et al., 2005; Williams et al., 2008; Peñaflor et al., 2011b; Tamiru et al., 2011; Michereff et al., 2019). Caterpillar-damaged plants are reported to be attractive to some *Trichogramma* spp. (Peñaflor et al., 2011b; War et al., 2016). Attraction to HIPVs is possibly important for some generalist *Trichogramma* parasitoids, when their host eggs and larvae co-occur (Peñaflor et al., 2011b; Michereff et al., 2019). Importantly, the volatile cues such as host pheromones, egg smells or even OIPVs are probably released with relatively smaller amounts compared to HIPVs, and then more likely working in a relatively short range (Peñaflor et al., 2011a; Michereff et al., 2016; Xu and Turlings, 2018). Therefore, HIPVs possibly facilitate host locations for some egg parasitoids in different ways.

Our data are in line with those that have been derived from the studies of some parasitoid species in rice (Liu et al., 2015), cotton (Moraes et al., 2011; Yao et al., 2016), and oilseed rape plants (Schuler et al., 1999, 2003). In maize, under treatment of caterpillar regurgitant, the *Bt* plant (N4640Bt) emitted fewer amounts of several volatile compounds than did its isogenic non-*Bt* line, but they showed similar attractiveness to two larval parasitoid species (Turlings et al., 2005).

Two major classes of HIPVs, green leaf volatiles and terpenoids, have often been considered to recruit natural enemies because they are the most abundant compounds emitted from plants after herbivore attack (Gershenzon and Dudareva, 2007; Arimura et al., 2009). However, some important volatile(s) emitted by maize plants responsible for attracting parasitoids are still not identified (D'Alessandro and Turlings, 2006; Turlings and Erb, 2018), though much effort has been made to identify them. For example, in maize plants, the attractiveness of HIPVs to the larval parasitoid *Cotesia marginiventris* (Cresson) probably relies on a combination of certain polar and non-polar compounds and the polar compounds are more important than the non-polar ones (D'Alessandro et al., 2009). However, the key attractants are normally released in trace amounts and even below the detection threshold of the GC analysis (Gouinguené et al., 2005; D'Alessandro et al., 2009). In contrast, some components of HIPVs emitted in large amounts (such as indole and some sesquiterpenes) do not attract natural enemies of herbivores (D'Alessandro et al., 2009; von Mérey et al., 2011). Those studies help us understand that in spite of occasionally changing the emitting amounts of some common VOCs in maize plants by transgenic events, the main attractiveness to parasitoids is possibly unchanged.

In conclusion, transformations of foreign genes to crops may change their VOCs emission. However, the variations are normally less apparent than those among conventional cultivars. Importantly, the modifications of VOCs emission normally do not reduce the attractiveness of GM plants to natural enemies. The findings indicate that releasing natural enemies is still likely to be an effective way to control non-target or *Bt*-resistant pests in *Bt* fields.

## Data Availability

The datasets generated for this study are available on request to the corresponding author.

## Author Contributions

JW, GC, BT, and HX conceived and designed the research. XW, GC, BT, and HX conducted experiments. GC, BT, and HX analyzed the data. HX wrote a first draft. All authors commented on the manuscript.

### Conflict of Interest Statement

The authors declare that the research was conducted in the absence of any commercial or financial relationships that could be construed as a potential conflict of interest.
