# Supplementary material for: Effects of Bacillus thuringiensis Genetic Engineering on Induced Volatile Organic Compounds Emission in Maize and the Attractiveness to a Parasitic Wasp
Source: Front Bioeng Biotechnol. 2019 Jul 10;7:160. doi: 10.3389/fbioe.2019.00160 (PMC6635655; doi:10.3389/fbioe.2019.00160)
Supplement: Supplementary file 2 [file Table_2.docx]

**Supplementary file 2** The amounts (ng/plant/4h) of volatiles released by different maize cultivars

| Compound^*^ | Intact | | | Artificial wounds + regurgitant | | | JA | | |
| --- | --- | --- | --- | --- | --- | --- | --- | --- | --- |
|  | 5422 | Bt1^△^ | Bt2^△^ | 5422 | Bt1 | Bt2 | 5422 | Bt1 | Bt2 |
| 1 | n.d.^◇^ | n.d. | n.d. | 4.3/0.8 | 6.3/1.3 | 7.5/2.6 | 6.7/1.6 | 8.8/3.9 | 17.6/3.4 |
| 2 | n.d. | n.d. | n.d. | 10.8/1.5 | 13.8/3.3 | 12.5/1.3 | 23.8/3.6 | 30.9/4.8 | 25.2/5.0 |
| 3 | n.d. | 8.7/0.8 | 5.8/0.9 | 40.8/5.1 | 72.6/13.9 | 62.3/11.3 | 98.6/8.8 | 164.9/7.0 | 71.7/5.3 |
| 4 | 18.7/8.9 | 17.2/5.6 | n.d. | 119.8/18.7 | 168.7/25.7 | 134.6/10.7 | 278.9/25.2 | 465.6/33.1 | 188.3/29.5 |
| 5 | n.d. | n.d. | n.d. | 8.6/2.0 | 12.7/2.3 | 9.1/1.1 | 22.3/4.4 | 32.1/6.1 | 26.1/6.9 |
| 6 | n.d. | n.d. | n.d. | 32.3/3.9 | 71.6/11.5 | 33.1/2.1 | 319.9/45.7 | 409.6/16.0 | 247.3/36.4 |
| 7 | n.d. | n.d. | n.d. | 4.2/1.1 | 8.8/1.8 | 3.8/1.5 | 22.2/4.9 | 22.2/9.9 | 12.7/0.9 |
| 8 | n.d. | n.d. | 5.8/1.4 | 6.3/2.0 | 10.9/1.7 | 7.0/0.9 | 26.8/4.5 | 18.9/4.1 | 11.9/1.9 |
| 9 | n.d. | n.d. | n.d. | 16.7/5.1 | 20.9/4.1 | 17.5/2.7 | 65.7/9.6 | 40.3/13.7 | 44.1/13.8 |
| 10 | n.d. | n.d. | n.d. | 10.0/1.5 | 10/1.4 | 13.1/0.8 | 57.4/7.7 | 61.8/8.8 | 39.7/10.6 |
| 11 | 4.5/1.5 | 19.4/6.9 | n.d. | 41.7/6.8 | 59.7/8.8 | 42.2/5.5 | 244.3/48.0 | 515.9/35.7 | 208.9/32.9 |
| 12 | n.d. | n.d. | n.d. | 7.1/1.1 | 8.4/1.2 | 8.0/1.2 | 71.9/6.3 | 98.2/13.4 | 75.9/5.8 |

*The amounts of compounds are presented as mean/SEM: 1 = (*Z*)-3-hexen-1-yl acetate, 2 = (*E*)-β-ocimene, 3 = linalool, 4 = (3*E*)-4,8-dimethyl-1,3,7-nonatriene (DMNT), 5 = phenethyl acetate, 6 = indole, 7 = methyl anthranilate, 8 = geranyl acetate, 9 = (*E*)-β-caryophyllene, 10 = (*E*)-α-bergamotene, 11 = (*E*)-β-farnesene and 12 = (*E*)-nerolidol; ^△^Bt1 and Bt2 are the transgenic *Bt* maize cultivars 5422Bt1 and 5422CBCL, respectively; ^◇^n.d. means the compounds have not been detected in our analyses.
